# Transcription factor Mohawk regulates tendon/ligament development: A narrative review

**DOI:** 10.1097/MD.0000000000043044

**Published:** 2025-07-25

**Authors:** Heng Yang, Chan Li, Qian Ding, Tai-Lai Li, Wei Tang, Hong-Jin Sui

**Affiliations:** a Department of Anatomy, Dalian Medical University, Dalian, Liaoning, China; b Department of Obstetrics and Gynecology, The Second Hospital of Dalian Medical University, Dalian, Liaoning, China; c The Second Affiliated Hospital of Dalian Medical University, Dalian, Liaoning, China.

**Keywords:** developmental regularity, mechanoforce, Mohawk (Mkx), tendon/ligament, therapy

## Abstract

Tendon and ligament injuries due to aging or overload are common clinical injuries of the locomotor system, often resulting in limited motion and pain. These diseases are difficult to partially cure because of their poor regeneration ability. Mohawk (Mkx) is a transcription factor that has been verified as critical to tendon/ligament development. Mkx knockout animals exhibit varying degrees of tendon defects, with multiple genes exhibiting different levels of expression. Mesenchymal stem cells and tendon stem/progenitor cells have been studied under circumstances of Mkx overexpression or deficiency, with or without mechanoforce stimulation. To further investigate the underlying mechanisms of tendon and ligament injury repair and develop therapeutic approaches, it is necessary to dig deeper into the molecular networks regulating tendon/ligament development. The study design is a narrative review. A search of the PubMed database was performed to conduct a comprehensive literature review on Mkx. A total of 119 studies were included. Recent studies have reported the importance of Mkx and its related genes on tendon/ligament developmental processes. In addition, numerous articles have also provided therapeutic aspects to Mkx-related tissue repair after injuries. Mkx plays an important role in tendon/ligament development, as well as the pathological processes. The combination of Mkx, Mkx-related molecular interaction networks with mesenchymal stem cells or tendon stem/progenitor cells, and 3-dimensioned cultural systems may offer a new thought for developing new strategies for acute and chronic tendon/ligament diseases.

## 1. Introduction

Tendons and ligaments are dense connective tissues that connect muscle to bone or skeleton.^[1–4]^ Their unique form conducts ability to transmit mechanical forces and enable locomotion. Differences exist though, they share similar gene expression profiles of their major cell type (tenocytes) and their structure.^[5]^ Damage or degeneration due to trauma or overuse could lead to various physical deterioration symptoms, such as tendon/ligament inflammation, rupture, regression, and so on. Tendons and ligaments have poor self-healing capacity, which may be partially attributed to their intrinsic trait of hypocellularity and hypovascularity.^[6]^ Currently available treatments are limited and secondary damage often occurs, at the site of lesion, the contralateral site, or the donor site.^[7–9]^ These lead to a series of research on regenerative therapy.

Mohawk (Mkx) is a recently discovered transcription factor that is reported to be tendon-specific and regulates tendon-related genes, such as Scleraxis (*Scx*) and tenomodulin (*Tnmd*).^[10–12]^ Over the years, studies have demonstrated that *Mkx* knockout mice showed a phenotype of systemic tendon defect^[11]^ and Mkx-induced mesenchymal stem cells (MSCs) could be used for tendon/ligament tissue repair.^[13,14]^

Therefore, this article is aimed to describe the common characteristics of tendon/ligament, as well as and the latest reports on the regulation of tendon/ligament development and healing responses by Mkx. Moreover, the role of Mkx in tissue engineering and its implications for basic and clinical research will be discussed. And finally, we will conclude with a summary.

## 2. Methods

A PubMed database search was conducted to gather studies on Mkx, tendon/ligament development, tendon/ligament developmental regularity, and tendon/ligament injury and therapy. The selection of articles was based on author-determined credibility, relevancy to the topic, and current trends in tendon/ligament development and injury repair. Moreover, additional articles were included in this review by referencing the bibliographies of previously obtained articles.

As a narrative review, the present study did not involve any type of human or animal testing and thus none of ethical approval is necessary.

## 3. Components, structure, and mechanical property of tendon/ligament

### 3.1. Components and structure of tendon/ligament

Responsible for the fundamental functions of force maintenance and transmission, water accounts for more than half of tendon/ligament total weight, whereas collagen accounts for the majority of the dry weight.^[15]^ Type I collagen, as a kind of structural collagen, is the predominant collagen of tendon and ligament, forming fibrils, accounting for approximately 90% and 85%, respectively.^[1,15]^ Non-fibrillar collagens, proteoglycans, elastin, etc together account for a small percentage, but they have a significant impact on molecular integration and biomechanical properties.^[1,3,15]^ These mentioned macromolecules, as well as minerals, together consist of a 3-dimensioned network known as an extracellular matrix (ECM).^[16–18]^ This spatially structured ECM provides structural and biochemical support to surrounding cells, providing the proper microenvironment for cell adhesion, cell communication, and cell differentiation.^[19,20]^

**Figure 1.**
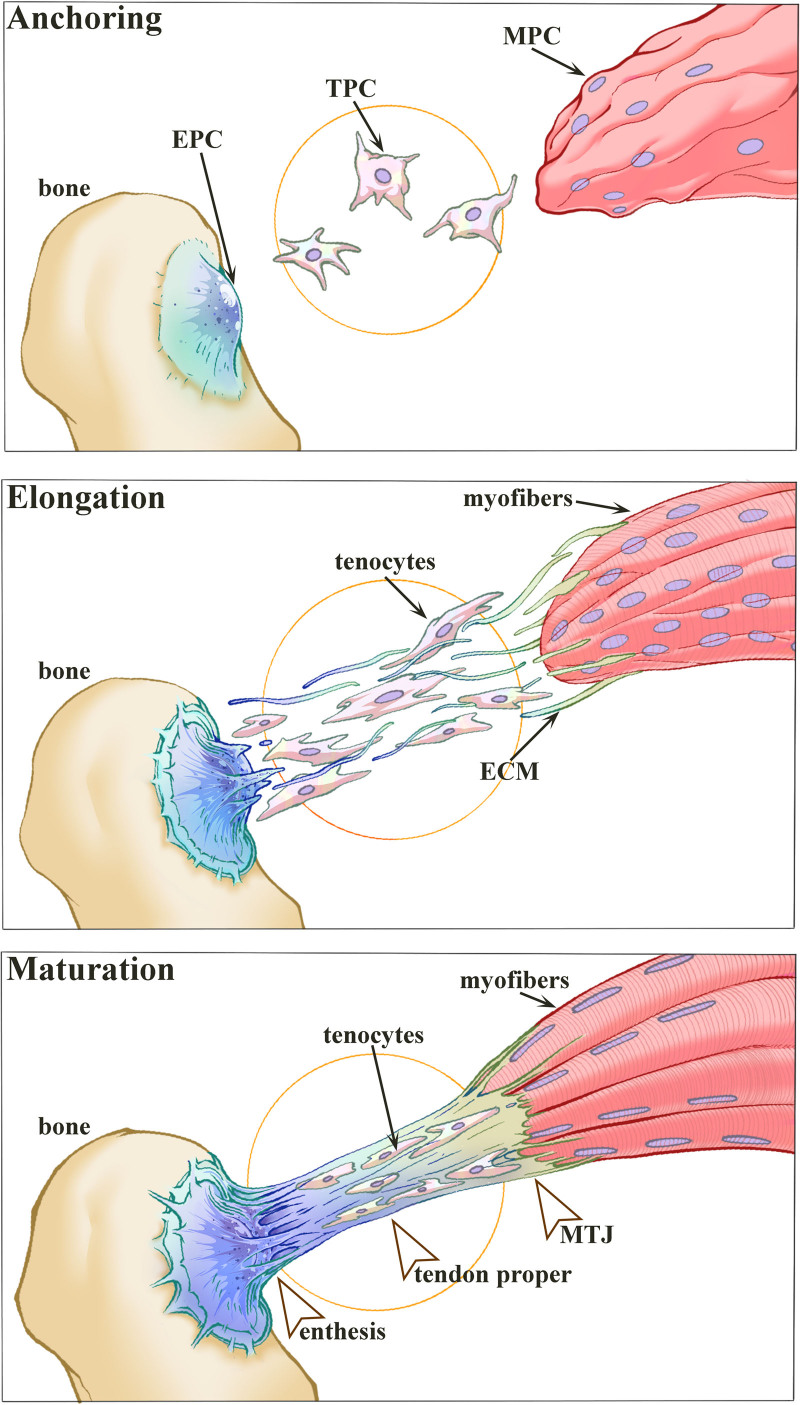
Schematic illustration of the current model of tendon development. The developmental phases are divided into the “Anchoring” phase, the “Elongation” phase, and the “Maturation” phase. ECM = extracellular matrix, EPC = enthesis progenitor cells, MPC = muscle progenitor cells, MTJ = myotendinous junction, TPC = tendon progenitor cells.

Tendon and ligament can be roughly divided into 2 parts, the body and the junction area. As for tendon, the junction area can be further divided into the osteotendinous junction and the myotendinous junction (MTJ),^[3]^ while ligament has only the former, which consists of a specialized interface known as enthesis.^[15,21]^ The bony attachments appear with unusual shapes, which in part shows how fibers are recruited during joint movement.^[15,21]^ MTJ is the transition area where sarcolemma and tendon collagen fibers are bound to each other through integration-related matrix molecules. The interface of MTJ is formed in an interfingering method in order to reduce local forces by increasing the interface area between them.^[15,22]^ The developmental phases of MTJ are generally divided into the “Anchoring” phase, the “Elongation” phase, and the “Maturation” phase. The “Anchoring” phase is a muscle-independent stage in which signaling induction from the cartilage plays the significant roles, while the “Elongation” phase is the stage of muscle-dependent, which is coordinated by stimulation of mechanical forces and bone growth; no muscle-dependent “elongation phase” occurs in ligaments. With the growth and development of the individual, the ligaments/tendons arrive the “Maturation” phase as well (Fig. 1).

**Figure 2.**
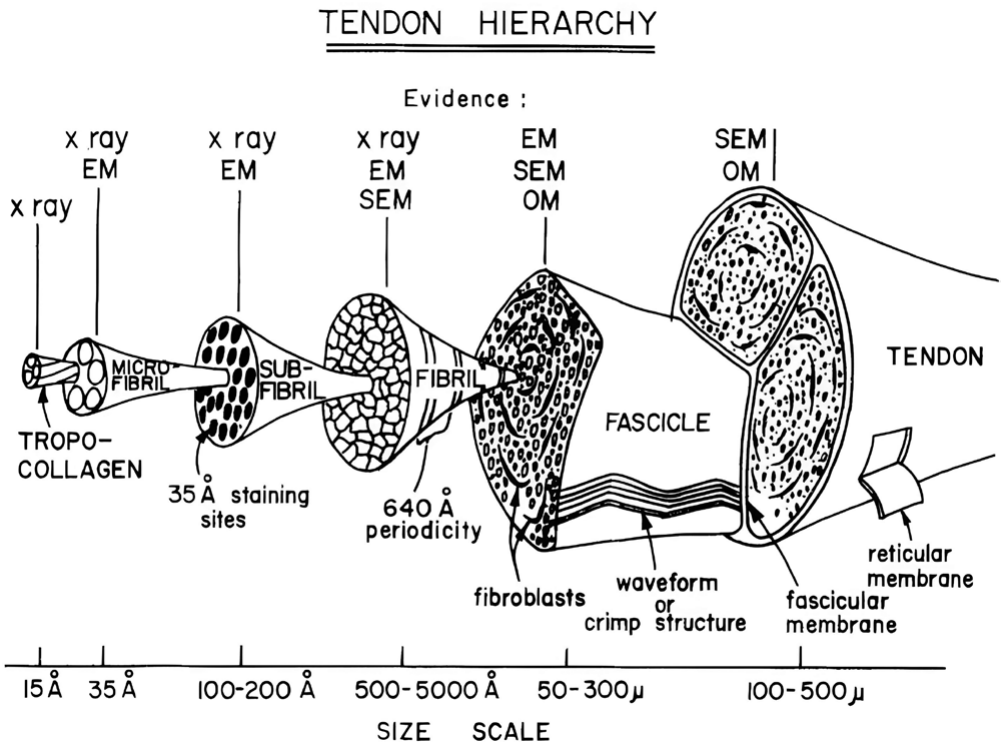
Tendon hierarchy (schematic diagram of the hierarchical structure of tendon/ligament tissue). Tendons and ligaments are hierarchical structures. Triple helical collagen molecules form the fibrils in a highly ordered manner. Bundled fibrils form the fibers and grouped fibers form the fascicles that are bundled by the endotenon. Tenocytes are located on the endotenon, connecting to one another through cellular channels. The epitenon is the fibrous sheath surrounding the tendon unit. Reused with permission from: Kastelic et al.^[23]^ The Creative Commons license does not apply to this content. Use of the material in any format is prohibited without written permission from the publisher, Taylor & Francis Ltd. Please visit www.tandfonline.com for further information.

Along with composition, the organization of tendons/ligaments determines their material properties. Taking tendons for example (Fig. 2), collagen molecules are the smallest building blocks and are aligned along the long axis of tendons.^[15,23]^ Grouped collagen molecules, in a highly ordered manner by intermolecular crosslinks, together form fibrils, fibers, and fascicles. Collagen fibers are not linear but presented in a “crimp” format, where the biomechanical function lies.^[2]^ Known as the primary structural unit, fibers can be seen under optical microscope while fascicle, the largest subunit of tendon, is visible to naked eye.^[15,23,24]^ Each fascicle is surrounded by a loose connective tissue known as interfascicular matrix (sometimes called endotenon). Continuous with interfascicular matrix, which binds fascicles together to form the complete tendon unit, is a connective tissue sheath covering the surface of tendon, so called epitenon.^[2,15,24]^ Endotenon and epitenon mainly consist of type III collagen and are thought to facilitate interbundle gliding during tendon movement.^[25]^

### 3.2. Mechanical property of tendon/ligament

**Figure 3.**
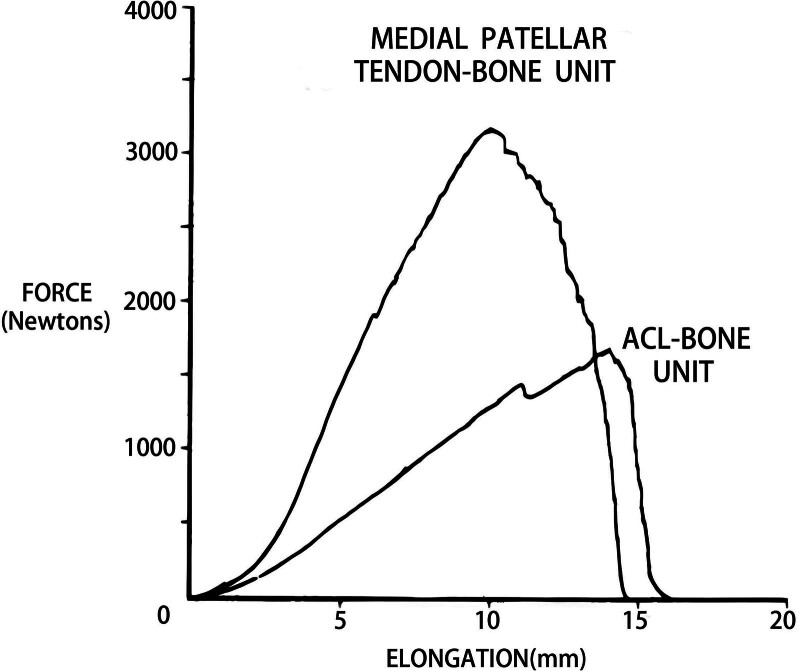
Load-elongation curve for tendons and ligaments, taking patellar tendon and anterior cruciate ligament as examples. Three distinct regions are presented in the curve by different line types as shown in the diagram. The lower left region of each curve is the nonlinear toe region that occurs on initial application of load. The following is the nearly linear regions over which the stiffness is measured. The area under the curve is referred to as the energy to failure. The original data stated in this figure was from Noyes et al.^[28]^ Reused with permission from: Noyes et al.^[28]^ The Creative Commons license does not apply to this content. Use of the material in any format is prohibited without written permission from the publisher, Wolters Kluwer Health, Inc. Please contact permissions@lww.com for further information.

As collagen-rich tissues, the hierarchical structure of tendons and ligaments determines their characteristics of relative compliance at low energy and low loading forces, while increasing stiffness with increasing force and load.^[26,27]^ The load-elongation curve demonstrates a triangular curve that can be divided into 3 regions, namely the toe region, the linear region, and the failure region (Fig. 3).^[28–30]^ In the toe region, that is, under low loading conditions, the crimped collagen fibers and viscoelastic properties are affected, and thus tendons and ligaments show relative compliance. While loading force increases, tendons and ligaments become stiffer. Depicted as the linear region, the curve is considered consistent with the slippage within collagen fibrils, then between fibrils, and finally reach the failure point, which is also known as ultimate load or point of ultimate tensile stress. And in the failure region, load continues to increase and at last causes a gradual avulsion between fibrils or an abrupt complete tear. Commonly, tendons and ligaments represent the same trend under the above tensile loading experiments, but the more detailed architectural differences affect their further mechanical characteristics. For example, the toe region is smaller in tendon because its more parallel collagen fibers require less realignment, while the linear region is larger in tendon due to its greater stiffness and tensile strength. These distinctions are clearly associated with their roles in organisms. Tendons undertake a more active role to mediate and maintain force transmission, whereas ligaments are considered passive but more flexible with the function of motion range control. The aforementioned properties dictate that tendons and ligaments may be injured in different situations. Tendons are likely to suffer from injury by overuse or sudden great force, yet ligaments are prone to shear forces.

In addition to usual tendon/ligament, various specific tendon/tendon structures exist, such as the annulus fibrosus (AF) of the intervertebral discs (IVDs), the periodontal ligament (PDL), or even the myodural bridge (MDB). IVD is important for spine movement by resisting stress, buffering stress, and increasing displacement. AF, especially the outer AF, mainly consisted of highly organized type I collagen, absorbing and balancing mechanical pressure to IVD along with nucleus pulposus.^[31]^ PDL is one of the periodontal tissues connecting teeth with the alveolar bone. The PDL resists and regulates the masticatory pressure to which the teeth are subjected and acts as a suspensory ligament, also known as the periodontium.^[32]^ MDB is a fibril connective tissue located between the suboccipital muscles and the cervical dura mater, passing through both the atlanto-occipital and the atlanto-axial interspaces in mammals and is mainly composed of type I collagen.^[33]^ Conducting force generated by suboccipital muscles to dura mater, MDB is speculated important for local cerebrospinal fluid flow.^[34,35]^ All these structures are not typical tendons or ligaments, but they have a similar collagen composition while assuming diverse biomechanical functions.

## 4. Introduction of Mkx

Mkx, also known as Irxl1 (Iroquois homeobox-like 1), is a homeobox protein encoded by *Mkx* (mohawk homeobox) gene, containing 353 amino acids.^[36,37]^ Mkx is a member of “three-amino acid loop extension” superclass of atypical homeobox proteins.^[36]^ Predicted orthologs in vertebrate and invertebrate species have been revealed by an iblastn search. Results showed that the homeodomain sequences were completely conserved at amino acid level among all examined vertebrates, while in invertebrates, the sequence homology was rather weak.^[36,38]^ The orthologs among vertebrates revealed Mkx a highly conservative and important protein during species evolution and individual development.

Gene expression analysis in mouse embryos has demonstrated that *Mkx* is expressed in tendons, muscles, cartilage progenitor cells, as well as male gonads and renal ureteric bud tip, during embryonic development, indicating an important role in regulating multiple developmental processes.^[39]^ The temporal-spatial traits have also been revealed in murine. Anderson et al^[36]^ studied the dynamic pattern of *Mkx* in mouse embryos, showing that *Mkx* transcription began at E9.0 in the dorsal region of the dermomyotome of the most-anterior somites and extended to the tail somites at E10.5. And at E12.5, *Mkx* was strongly expressed in autopods. Despite being widely expressed, *Mkx* knockout mice did not have significant defects in muscle, cartilage, and bone but rather a systematic tendon and ligament defect.^[11]^ This displayed a strong association between Mkx and tendon/ligament development. Later, Suzuki et al^[40]^ found similar general tendon hypoplasia as well as heterotopic ossification of the Achilles tendon in rats.

Recent research has focused on how Mkx regulated tendon/ligament development and has displayed that Mkx was an important transcription factor.^[37,41]^ Both in vitro and in vivo studies demonstrated the ability of Mkx to induce tenogenic differentiation.^[42]^ Deficiency and overexpression of *Mkx*, which could be caused by illness or degeneration or specific mechanical stimulation clinically, functioned differently on tenogenic, chondrogenic, osteogenic, and adipogenic process.^[36,40,43]^

## 5. Effects of Mkx on tendon/ligament

### 5.1. Phenotype of Mkx^−/−^ animals

Based on the temporal-spatial variation of *Mkx* in mouse embryos, Ito et al^[11]^ and Liu et al^[12]^ studied the in vivo function of Mkx in developing tendons. By generating *Mkx* mutant mice, they observed a distinctive wavy-tail phenotype due to tendon hypoplasia. A close examination was performed and found that the tendon hypoplasia was a generalized phenotype, but not a partial one. Despite the apparent reduction in tendon mass and size, the cell counts in tail tendon fiber bundles of *Mkx* null mice were not significantly different from those of wild-type mice. This suggested the reduction of ECM synthetic ability or the enhancement of ECM catabolic activity. Further experiments then revealed that the level of type I collagen, the main component of tendon ECM, was downregulated in *Mkx*^−/−^ mice. According to these studies, Mkx does not affect the initiation of tendon formation because cell numbers do not show differences, but considering the changes in tendon ECM, Mkx affects tendon differentiation and maturation. In consideration of the limited biomechanical property of the small size, studies have been conducted on rats. *Mkx* knockout rats generated by CRISPER/Cas9 method presented early heterotopic ossification of the Achilles tendon in addition to systematic tendon defect.^[40]^ In vitro experiments of mechanical strains revealed that mechanical stretch stimulation led to chondrogenic differentiation in *Mkx*^−/−^ tendon-derived cells (TDCs) but resulted in tenogenic differentiation in *Mkx*^+/+^ TDCs. Further rescue experiments showed that chondrogenic, osteogenic, and adipogenic differentiation of *Mkx*^−/−^ TDCs could be suppressed by overexpressing *Mkx*. The above experiments all contributed to tendon maturation and property maintenance in the Mkx context.

In addition to mammals, experiments were also performed on zebrafish out of consideration for perfect homology of Mkx homology domains among vertebrates. Iroquois homeobox-like 1 knockout zebrafish exhibit a flattened and small head, shortened and curved body axes and tails, shrunken yolk stalk, and circling swimming behavior.^[44,45]^ Whole-mount in situ hybridization and immunofluorescence staining revealed the importance of *Mkx* in brain region formation and segmentation during brain development, as well as in pharyngeal arch development and myotome formation during muscle differentiation. And myotome formation was especially related to myoseptum formation, scilicet the tendon element in fish body trunk.^[46]^

To sum up, Mkx is important during tendon/ligament structure development among the above vertebrates, indicating a potential role for Mkx in related signaling pathways.

### 5.2. Tendon/ligament developmental regularity

#### 5.2.1. Molecules and signaling pathways

**Figure 4.**
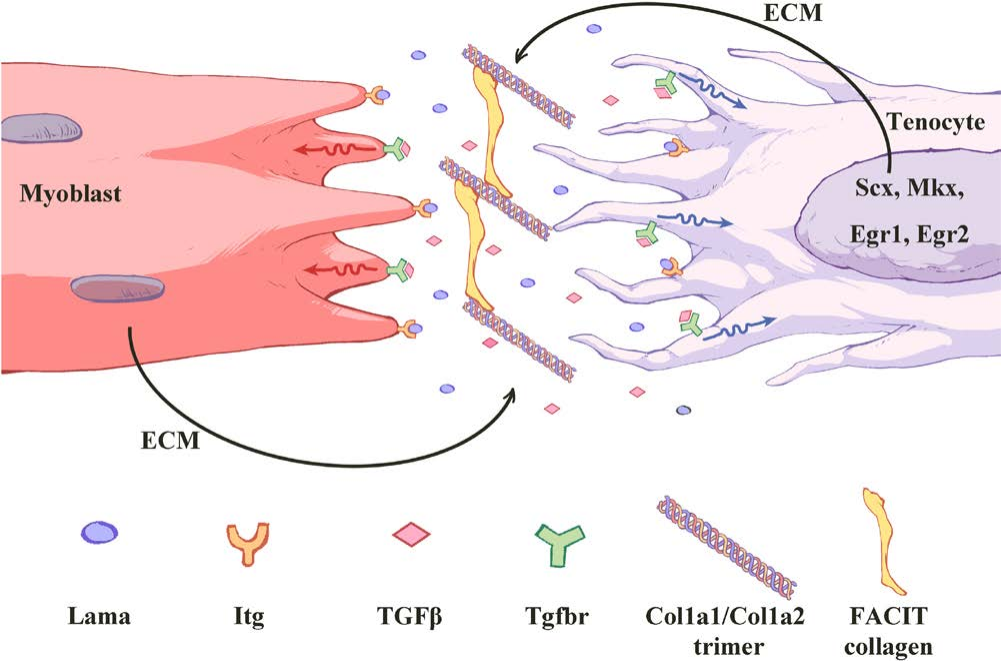
Schematic illustration of tendon development regularity. The main types of ECM of tendon, including Col1a (triple helix), Lama (purple), etc, are synthesized by tenocytes. Myoblast also synthesizes and secretes some ECM such as Col22a1 (belonging to FACIT collagen (yellow)). The signal responses to mechanical stress (wavy line arrows) through Itg receptors (orange) on myoblast and tenocyte cell surfaces. Stress causes the ECM to release TGFβ (pink) from the TGFβ large latent complex (LLC). Itg and TGFβ signaling in tenocytes feedback to regulate Scx-, Egr1/2-, and Mkx-induced transcription to modulate tendon stiffness. ECM = extracellular matrix, Egr1/2 = early growth response 1/2, Mkx = Mohawk, Scx = scleraxis, TGFβ = transforming growth factor beta.

Multiple genes and proteins participate in tendon/ligament formation and homeostasis, but not as many have been adequately studied. Here we focus on Mkx and several relevant molecules that could respond to changes in Mkx levels (Fig. 4).^[22]^

Scx is the first transcription factor described to regulate tendon development.^[47,48]^ As the earliest recognized marker of tenocyte stem/progenitor cells, its expression was detected between sclerotome and myotome at E9.5 and subsequently in syndetome.^[25,47,49]^ Induced by the interaction of sonic hedgehog and fibroblast growth factor (FGF) signaling, *Scx* was expressed in the syndetome area, and by transforming growth factor beta (TGFβ) signaling in the limbs.^[48–50]^ In vitro experiments yielded differentiation from MSCs and human embryonic stem cells to tendon cells by *Scx* overexpression.^[51,52]^ *Scx*-null mice represented force-transmitting tendon atrophy and tendon ECM disorganization.^[53]^ As a transcription factor, Scx, along with Nfatc4, bound to cis element of type I collagen to activate the *Col1a1* gene specifically in tendon fibroblasts.^[54]^ Furthermore, Shukunami et al^[55,56]^ reported that Scx could induce the expression of *Tnmd* in a tendon cell lineage-dependent manner. Tnmd, considered a late marker of tendon/ligament development, is a type II transmembrane glycoprotein that is predominantly expressed in tendons and ligaments.^[55]^ Tnmd deficiency caused tendon premature senescence in mice, though key tendon-related markers showed no differences.^[57]^ Also, tendon elasticity increased in *Tnmd* mutant mice, indicating functional characteristics changing.^[58]^ These results showed that Scx is a vital transcription factor involved in tendon differentiation and development, partly by regulating tendon-related markers like Col1a1 and Tnmd.

Early growth response 1/2 (Egr1/2) are zinc finger transcription factors reported critical for tendon formation.^[59]^ Taking mouse embryogenesis as an example, *Egr1* was expressed in the syndetome at E12.5 and Egr2 was transiently expressed in tendons at E14.5.^[60]^ In immediate response to FGF4 stimulation, the expression of *Egr1* and *Egr2* was enhanced, followed by enhanced expression of *Scx*, *Col1a1*, and other tendon-related collagens.^[59,60]^ *Egr1* knockout mice exhibited a reduction of collagen fibril number in tendons as well as a reduced expression of multiple tendon-related genes, such as *Scx* and *Mkx*.^[60]^ It should be noted that in *Egr1*^−/−^ mice, reduced expression of *Scx* persisted into adulthood, while that of *Mkx* was restored, showing a transformation of *Egr1* expression pattern among developmental and adulthood.^[61]^ TGFβ signaling was activated in *Egr1* overexpressing MSCs and gave rise to Scx upregulation.

As mentioned above, Mkx is a transcription factor generally expressed in tendon and ligament tissue.^[41]^ The expression pattern of *Mkx* is later than that of *Scx* and *Egr1*, suggesting that it is not required for tendon formation during the initial phase.^[36]^ At first, *Mkx* null mice from normal tendons had no defects in *Scx* expression, but later exhibit reduced diameters of collagen fibrils and reduced levels of relevant genes like *Col1a1*, *Tnmd*, fibromodulin, and decorin.^[11,12,62]^ Mkx has a dual function during tenogenic phase. Functioning as a repressor, Mkx represses myogenesis, forming a complex with sin3A/histone deacetylase, by suppressing *MyoD* and *Sox6* expression as well as represses skeletogenic fates of tenocytes by suppressing *Sox9* and *Runx2* expression.^[40,41,45,63]^ Translating the effect into an activator, *Mkx* overexpressing elevated the level of *Scx* and *Col1a1* in either human MSCs or C3H10T1/2 cell lineage.^[10,42]^ Liu et al,^[14]^ using chromatin immunoprecipitation sequencing assays, revealed that Mkx upregulated Scx by directly activating *Tgfb2*, indicating that it regulated tenogenesis process partially via TGFβ signaling pathway. It is notable that tendon stem/progenitor cells (TSPCs) are still developing even when *Scx* or *Mkx* or *Egr1* are knockout, suggesting that none of these discovered molecules is decisive for tendon differentiation to date. But the interactions among them are important for tenocyte differentiation and character maintenance as well as ECM production and assembly.

Matrix metalloproteinases (MMPs) are proteinases that could degrade almost all ECM proteins along with the ability to remodel them.^[64]^ Tissue inhibitors of metalloproteinases act synergistically with MMPs for maintaining local homeostasis by strictly controlling the involved enzymes to balance the degradation process.^[64]^ Studies have shown that disturbed *MMPs* expression is related to tendinopathy and osteoarthritis, including *MMP-2*, *MMP-3*, *MMP-8*, *MMP-9*, etc.^[65–67]^ Recent research on diabetic tendinopathy has demonstrated a significant decrease of COL1A1, which is associated with the downregulation of tenogenic marker genes *Scx* and *Mkx*, and with upregulation of ECM catabolic genes *MMP-9* and *MMP-13*.^[68]^ This may not be evidence of a direct relationship between Mkx and MMPs, but it is certain that both are involved in tendon ECM metabolism.

Except for embryogenesis period, tendon continues developing postnatally. Conditionally knockout regulatory-associated protein of mTOR (Raptor) built a mTORC1 function-loss postnatal mouse model that showed a significantly decreased tendon thickness but still a number of tendon cells.^[69]^ In contrast, mTORC1 activated mouse model by *Tsc1* deletion revealed increased tendon cell number and proliferation, as well as disorganized fibers, hypercellularity, and neovascularization, However, there was little change in tendon thickness.^[69]^ Both inhibition and activation of mTORC1 signaling affected generation of collagen fibrils.^[69]^ Collectively, these results demonstrated that properly regulated mTORC1 signaling is required during postnatal tendon maturation.

Since *Mkx* was discovered later than *Scx*, the upstream signaling pathway that regulates Mkx has not been well studied. Except TGFβ signaling, RNA-seq of Achilles tendon from mTORC1 loss-of-function mice showed decreased expression of *Mkx*, as well as *Tnmd*, *Col1a1*, decorin, and fibromodulin.^[69,70]^ These results represent a correlation between Mkx and mTORC1, and further studies are needed to uncover how they interact with each other and with other signaling pathways.

In recent years, single-cell transcriptomic analyses have been utilized to study the cellular heterogeneity of mouse Achilles tendon and 11 distinct types of cells, including 3 populations of tendon fibroblasts that had not been discovered ever before, were uncovered.^[71]^ Together with trajectory inference analysis, the researchers provided evidence for the previous view that pericytes in the tendon vascular system are a potential adult tendon fibroblast (or at least one of the populations) progenitor cell source.^[71]^ Based on these, an interactive tendon atlas was built to identify the heterogeneity between and within tendon cell populations.^[71]^

#### 5.2.2. Mechanical stress and signaling

Considering tendon/ligament functions of force transmission and position stabilization, muscle contributes significantly to force generation and joint displacement. Taking distinct development patterns of axial muscle and limb muscle into account, muscle development is essential in the early stages of axial tendon formation and in the later stages of extremity tendon elongation.^[72–74]^ During embryogenesis, TSPCs and muscle progenitor cells first co-locate and then align in the direction of muscle contraction. Muscle-transported ECM is involved in the formation of the finger fold MTJ, collaborating with tendon-derived ECM through multiple pathways (e.g., EGF signaling, FGF signaling, and retinoic acid signaling).^[75–77]^

Studies showed that proper exercise load could increase type I collagen amount of tendon by increasing TGFβ level and maintain Scx level through TGFβ/Smad2/3 pathway, indicating that organisms transmitted mechanoforces into biochemical signals often by TGFβ signaling pathway.^[78–80]^ This is also the way tenocytes and TSPCs respond to mechanical loading. Besides, cyclic uniaxial mechanical loading could increase the expression of *Scx*, *Egr1*, *Col1a2*, etc in teno-induced pluripotent stem cells.^[43]^ Mechanosensory systems in tendon cells and TSPC are also thought to be critical in these events. Moreover, experts conducted experiments to understand the adaptation and response of muscle-tendon units under plyometric training.^[81]^ The tests showed that muscle architecture, tendon structure, muscle–tendon stiffness, and physical performance all changed after plyometric training program in varying degrees and mitogen-activated protein kinase signaling was activated specific to eccentric rather than concentric training, indicating strong plasticity of muscle and tendon in the face of diverse mechanoforce.^[81,82]^ In physiological conditions, there are adaptive changes in muscles and tendons to various movement patterns.^[82]^ After concentric and eccentric training, a shift in MTJ position and muscle bundle angle was found.^[82]^ Besides, a study showed that fast-running training could upregulate *Tgfb* expression in accordance with type I collagen deposition, suggesting the adaptation of ECM to mechanical loading.^[83]^

Focusing on Mkx, Smad3, a downstream molecule of TGFβ signaling pathway, could interact with Mkx to form a complex, representing that Smad3 may enhance Mkx function during tenogenesis.^[25,84]^ Moreover, Mkx responded to mechanoforces both in vivo and in vitro partly through general transcription factor II-I repeat domain-containing protein 1 (Gtf2ird1).^[85]^ Gtf2ird1 presented nuclear translocation when exposed to cyclic mechanical stretching and its knockdown showed no increased activity of *Mkx*.^[85]^ These results revealed that the Smad3/Mkx complex and nuclear translocation of Gtf2ird1, a kind of mechanosensory in tenocytes, were both essential for *Mkx* transcription and function (e.g., Mkx-mediated tendon homeostasis and regeneration).

### 5.3. Tendon/ligament tissue healing

Three overlapping phases have been described after acute tendon injury, known as inflammation, proliferation, and remodeling phase chronologically.^[6,86]^ The healing process is closely linked to vasculature. This can manifest itself as avascular tendons/ligaments, such as rotator cuff and intra-articular ligaments, with little to no self-healing capacity.^[87,88]^ Tendon tissue repair begins with local clot formation, followed by activation of inflammatory cells and finally fibroblast recruitment.^[89]^ The local inflammation is caused by cytokines such as TGFβ and insulin-like growth factor-1, and platelet-derived growth factor, which are released by platelets and cells.^[90,91]^ Three characteristics are recognized as the features of the proliferation phase, namely ECM expansion, cellularity increase, and fibrovascular scar deposition.^[89,92]^ Considering that collagen synthesis is an oxygen consumption process, angiogenesis is significant so that oxygen, nutrients, and growth factors, could be offered to the surrounding cells.^[93]^ Vascular endothelial growth factor and basic FGF are highly expressed to promote angiogenesis and cellular proliferation during this period.^[94,95]^ Remodeling usually begins 2 weeks after injury and continues for months or even years, with collagen reorganization in the same direction as the stress.^[86]^ At the lesion, type III collagen is absorbed as the vascular, cellular, and water content decreases.^[92]^ Type III collagen is gradually replaced by type I collagen, which accounts for a higher percentage of the tensile strength.^[89]^

The process of tendon/ligament healing is a typical incomplete regeneration, also known as scar repair.^[96,97]^ Though the tissues do repair themselves, they are unable to restore the structure and function of the original tissue.^[88]^ That is why even the vascularized tendons and ligaments cannot recover their ultrastructural and biomechanical properties from injury.^[87]^ Thus, researchers and clinicians devoted to developing regenerative therapies to help patients reconstruct tendon and ligament function as much as they can.

## 6. Therapeutic approaches and Mkx-related tissue engineering

*Mkx* is critical not only in tendon/ligament formation and maturation, but also in tendon/ligament tissue healing and repair. *Mkx* is highly expressed in TSPCs compared to other progenitor cells like adipose stem cells, and embryonic stem cell-MSCs.^[14]^ Inducing committed differentiation to tendon/ligament cells, *Mkx* overexpression could impair MSCs differentiation to adipogenic and osteogenic lineages.^[14]^ Function execution of Mkx, like the ability to induce other factors, is diversified according to different cell lines. Using adenovirus vector transduction to overexpress Mkx protein in human bone marrow-derived mesenchymal stromal cells, tenogenic related proteins Col1a1, Tnxb, Tnc, and Tnmd exhibited a remarkable increase, while *Scx* was not activated.^[42]^ However, in mouse tail TSPCs and mouse MSC cell line C3H10T1/2, *Col1a1*, *Tnc*, *Tnmd*, as well as *Scx* were all induced and presented high expression level.^[14]^ Neither human bone marrow-derived mesenchymal stromal cells nor C3H10T1/2 displayed an increase of *Egr1* upon *Mkx* induction.^[14,42]^ The different species origin and cell types of stem cells may be responsible for these differences.^[38]^ For basic studies, integration of the transcription factor Mkx and multiple cell lines allows for learning key information at a deeper level about developmental patterns and repair processes.

Clinically, methods have been developed to deal with tendon/ligament injuries. Generally, tendon/ligament injuries are treated with conservative management, surgical methods, or stem cells.^[86,98,99]^ Physical therapies and biologics injections are utilized as normal conservative management. The most often used biologics are hyaluronic acid and platelet-rich plasma (PRP).^[100,101]^ Clinical studies have shown that, based on its biocompatibility, viscoelasticity, and other physicochemical characteristics, hyaluronic acid has been recommended for application in tendinopathy.^[102]^ PRP contains multiple growth factors, including but not limited to platelet-derived growth factor, vascular endothelial growth factor, TGFβ, EGF, FGF, and insulin-like growth factor.^[86,103]^ Though there is no high-level evidence for PRP use, PRP injection remains a viable strategy for tendon or ligament damage, which may be attributed in part to its low risk of complications.^[104]^ Conservative management are conducted not only as a single therapeutic strategy, but also as part of pre- and post-operation treatment. Surgical methods are common for tendon/ligament injury, especially rupture with avascular tendon/ligament. The effectiveness of simple suture depends on the conditions of damaged area, the type of stitches, as well as the suture technique.^[105,106]^ When faced with a tissue defect, a graft may be chosen depending on the condition, which can be further classified as autograft, allograft, and xenograft.^[107,108]^ The former one concerns the donor site morbidity while the latter 2 refer to the availability of tissue, the risk of immunogenic response, and the risk of pathogen infection.^[108,109]^ With the progression of tissue engineering, multifarious other grafts become known to the public, including scaffold, stem cell, and cell secretory product related engineering.^[110,111]^ Local injection of growth factors has been conducted over decades. Such attempts have shown significant efficacy for the early stage of tendon and ligament healing but limited long-term efficacy.^[91,112]^ Meanwhile, MSCs from various sources have been utilized for tendon and ligament tissue repair. Numerous studies have demonstrated the effectiveness of stem cell strategy, both in vitro and in vivo. With the development of tissue engineering, despite simple injection, stem cells combining scaffold or transcriptional factors or growth factors are gaining importance. Multiple factors have been endeavored to observe the stimulation to MSCs or TSPCs, and so has Mkx.^[40,109,113]^ Mkx induces differentiation of MSCs toward tendons and inhibits differentiation of MSCs toward other lineages, including osteogenesis, chondrogenesis, and adipogenesis.^[10]^ Mechanical stress stimulation also takes part in *Mkx* expression regulation.^[40,85]^ Therefore, it can contribute to clinical application when integrated with MSCs. MSCs can provide tendon/ligament the capacity of self-renew and the potential of differentiation while Mkx can directionally induce MSCs into tenocytes and maintain their tendinous characteristics, which contributes to long-term efficacy.^[14,114]^ Additionally 3-dimensional culture may be added to offer oriented ECM and better tissue ingrowth.^[115]^ These should be further studied in the near future.

## 7. Conclusion and future direction

With the progressive discovery of critical transcription factors in tendon and ligament development, the development and mechanical mechanisms have been uncovered. Mkx is a transcription factor regulating tendon/ligament development and maintaining tendon/ligament homeostasis. Participating in mechanical force response, Mkx is also closely related to tendon/ligament injury. Animal experiments have already utilized Mkx to study its role in tissue repair, with the participation of multiple MSCs or mechanoforces. Treatment strategy for tendon and ligament injury still obsesses clinicians and researchers, with physical and surgical treatments remaining the only options. With the development of tissue engineering as well as the developing understanding of tendon/ligament development and healing, efforts will be devoted to throwing light on the function of transcriptional networks and how to develop biodegradable and biocompatible grafts with adequate strength and durability. Meanwhile, as a functional whole, different parts of motor system interdepend and coordinate on each other throughout development.^[116,117]^ Bone dynamics and homeostasis is coordinately related to osteoclasts, osteoblasts, osteocytes, and chondrocytes with their respective transcription factors. Such as Dmp1, Phex, and sclerostin for osteocytes; Dlx5 for chondrocytes; NFATc1 for osteoclast; and osteocalcin, sclerostin, and Runx2 for osteoblasts. For example, Osterix has several functions in these cells separated.^[118]^ Furthermore, a study in 2021 revealed that abnormal development of MTJ can lead to decreased bone mass.^[119]^ Therefore, the role of Mkx in other cell types such as osteoblasts also needs to be further studied. Although the major focus of the present study is the regulatory roles of Mkx in tendon/ligament development, it is also hoped to arouse the research interests of Mkx’s interactions with other cell types or tissues.

## Author contributions

**Conceptualization:** Heng Yang, Chan Li, Hong-Jin Sui.

**Supervision:** Chan Li, Hong-Jin Sui.

**Validation:** Qian Ding, Wei Tang.

**Visualization:** Heng Yang, Tai-Lai Li.

**Writing – original draft:** Heng Yang.

**Writing – review & editing:** Heng Yang, Chan Li.
